# Medical and Surgical Emergencies in Occupational Medicine: A Comprehensive Review

**DOI:** 10.7759/cureus.76985

**Published:** 2025-01-06

**Authors:** Joshua A Jogie, Jeremy Jogie, Amrita P Ramharacksingh, Nyeil C Ali

**Affiliations:** 1 Faculty of Medical Sciences, The University of the West Indies, St. Augustine, TTO; 2 Family Medicine, Chaguanas District Health Facility, Chaguanas, TTO; 3 Accident and Emergency, North West Regional Health Authority, Port of Spain, TTO

**Keywords:** emergency management, injury prevention, occupational medicine, trauma, workplace hazards

## Abstract

This review aims to provide a general resource for occupational health stakeholders. It also serves as a clinical guide for frontline providers and a policy framework for employers and regulators. Medical and surgical emergencies in occupational settings can cause serious harm if not identified and managed early. This review covers trauma, chemical exposures, thermal injuries, respiratory distress, and infectious hazards. We outline clinical signs, diagnostic steps, initial care, and follow-up plans. We also discuss preventive strategies such as hazard assessments and safety protocols. Evidence-based guidelines and practical methods can lower injury and death rates. Our objective is to help stakeholders recognize risks, respond fast, and improve outcomes. Future studies should monitor and examine new threats, such as novel industrial processes and evolving pathogens, to optimize workplace safety.

## Introduction and background

Occupational medicine studies diseases and injuries from workplace exposures, processes, and environments. It draws on toxicology, epidemiology, and clinical medicine. Workers in many sectors face serious risks from chemicals, physical hazards, and psychosocial stressors. Understanding acute medical and surgical emergencies in the workplace is important, as these events often demand immediate action. Table 1 summarizes major types of occupational hazards and their common outcomes.

**Table 1:** Common occupational hazards and typical injuries CNS: central nervous system

Hazard type	Examples	Typical injuries or illnesses
Trauma	Falls, machinery malfunctions, crushes	Fractures, hemorrhage, amputations, blast injuries
Chemical exposures	Toxic fumes, heavy metals, pesticides	Respiratory distress, organ failure, CNS effects
Thermal injuries	Burns from hot surfaces, open flames	Superficial to deep burns, shock, fluid loss
Respiratory hazards	Dust, fibers, gas leaks	Chemical pneumonitis, wheezing, pulmonary edema
Infectious hazards	Needlestick, zoonotic infections	Viral or bacterial illnesses, blood-borne pathogens
Ergonomic hazards	Improper lifting, repetitive tasks	Acute musculoskeletal strain, nerve compression
Psychological hazards	Stress, burnout, traumatic incidents	Panic attacks, anxiety, risk of self-harm

Traumatic workplace injuries range from superficial wounds to life-threatening events [1]. Falls, vehicle collisions, and machinery accidents contribute to blunt or penetrating trauma [2]. Heavy machinery can cause crush or amputation injuries. Explosives may lead to complex blast wounds involving bones, vessels, and soft tissue [3]. Rapid first aid and evacuation are critical in these instances. Providers must consider contaminated wounds that involve chemicals or pathogens [4].

Chemical exposures pose acute risks. Inhaled or dermal contact with solvents, pesticides, or heavy metals can cause respiratory distress, organ toxicity, and other systemic effects [5]. Industries handling reactive chemicals face possible fire outbreaks, explosions, or toxic releases [6]. Immediate evacuation, substance identification, and targeted treatments are vital. Antidotes like chelating agents can help, but success ultimately depends on quick triage.

Thermal injuries are also common. Workers exposed to molten metals, open flames, or high temperatures face burn-related risks [7]. Burns range from first-degree to severe third-degree injuries. Electrical burns can damage deep tissues with minimal surface signs, while chemical burns from strong acids or alkalis create unique management challenges [8]. Early burn care, fluid management, and possible burn unit referral improve outcomes.

Respiratory emergencies occur in dusty or chemical-heavy industries [9]. Large dust loads can lead to acute breathing problems. Toxic gas leaks (e.g., ammonia, chlorine) can cause immediate airway irritation, wheezing, or pulmonary edema [10]. Removal from exposure and providing supportive therapies, such as oxygen or bronchodilators, are key. Proper ventilation, sealed storage, and personal protective equipment (PPE) help prevent these events [11].

Infectious hazards affect certain occupations. Needlestick injuries can transmit hepatitis B, hepatitis C, or HIV [12]. Laboratory workers are at risk of exposure to pathogens. Farmworkers may face zoonotic diseases. Occupational context often guides testing and prophylaxis. Fast recognition and management including immunoglobulin or antiretroviral therapy can prevent severe outcomes [13]. Employers must maintain protocols for hazardous materials and biosafety. However, outbreaks still occur when guidelines fail [14].

Ergonomic hazards can prompt sudden emergencies. Improper lifting might cause acute back injuries. Repetitive tasks can lead to nerve compression or tendon rupture [15]. Proper training, breaks, and mechanical aids help reduce these incidents. Once an acute injury occurs, prompt imaging and specialized care can limit disability.

Psychological emergencies are also reported in workplaces. Stress, burnout, or traumatic incidents can trigger panic or other crises [16]. High-stress fields see higher suicide rates, often linked to chronic strain or easy access to lethal means [17]. Employers should actively promote mental health resources. Early support and crisis intervention can prevent severe consequences [18].

Disasters, such as natural events or large-scale industrial accidents, can cause mass casualties [19]. Multiple injuries (e.g., burns, crush trauma, chemical exposures) may overwhelm local healthcare resources and authorities. Coordinated rescue, containment, and triage are paramount in such scenarios. Surge capacity, mass casualty protocols, and well-rehearsed drills can help reduce morbidity [20].

Government agencies like the Occupational Safety and Health Administration (OSHA) set permissible exposure limits (PELs) and mandate training [21]. Enforcement varies, and some workplaces struggle to comply. Even in well-regulated settings, accidents happen from human error or unpredictable events. Continuous monitoring, inspections, and penalties push employers to put in place efforts to improve safety [22]. New technologies can introduce unrecognized hazards. Robotics may reduce repetitive strain but cause injuries if they malfunction [23]. Nanotechnology raises concerns about the inhalation of microscopic particles. Novel chemicals in advanced batteries may be toxic [24]. Clinicians must stay alert to unusual presentations that might point to emerging workplace risks.

Workplace diversity affects emergency outcomes. Differences in language, culture, health literacy, and preexisting conditions can complicate prevention and response [25]. Older workers or those with chronic diseases may be more vulnerable in crisis situations [26]. Clear communication and inclusive training promote better outcomes. Economic constraints also shape safety. Smaller businesses may lack funding for protective measures or advanced training. Underreporting of hazards can skew data and hamper effective interventions [27]. Temporary or contract workers often have fewer protections. Socioeconomic factors can widen disparities in occupational health.

Translating research into clinical practice can be slow [28]. New providers may have limited training in occupational medicine. Continued education and practical partnerships with local industries help fill knowledge gaps. Professional organizations distribute practice guidelines. Integrating them into clinical workflows can expedite adoption [29]. Table 2 lists some strategies to bridge the gaps between research and practice.

**Table 2:** Strategies to bridge the gaps between research and clinical practice

Strategy	Examples
Continuing education	Workshops, online modules, conferences
Local industry partnerships	Sharing chemical inventories, hazard alerts
Guideline integration	Embedding protocols in electronic health records
Ongoing surveillance	Real-time reporting of injuries and near-misses
Rapid data exchange	Telemedicine consultations, poison control collaboration

Surveillance systems that track injuries and illnesses guide prevention. These data reveal patterns and high-risk sectors [30]. Major incidents in the past, such as factory fires, have spurred major reforms related to workplace safety [31]. But modern workplaces are more complex. The coronavirus disease 2019 (COVID-19) pandemic showed how infectious threats can spread quickly and disrupt industries [32]. This event forced many organizations to adopt new safety measures.

Communication is vital for preventing and managing emergencies. Clear labels, ongoing drills, and mentoring help workers to be alert and aware of the actions to be taken in a crisis [33]. Investigating near-misses offers a chance to fix system flaws before serious harm occurs. Proper first aid training, basic life support tools (e.g., AEDs, eyewash stations), and specialized equipment can save lives [34]. Collaboration with external responders improves triage and treatment [35]. Table 3 highlights key immediate-response actions.

**Table 3:** Key steps in immediate occupational emergency response EMS: emergency medical services

Step	Action
Early hazard recognition	Identify the source (chemical leak, gas, mechanical failure)
Rapid first aid	Stop bleeding, stabilize fractures, cool burns, administer O_2_
Contain or isolate hazard	Turn off machinery, close valves, use spill kits
Call for external support	Contact EMS, fire department, poison control center, if needed
Evacuation and triage	Move workers to safety, prioritize severe injuries
Notify onsite leadership	Activate internal emergency plan, document incident

After undergoing acute care, employees returning to work require fit-for-duty assessments [36]. Early or inappropriate returns may lead to re-injury. Mental health follow-up is also important. Employers can modify tasks or schedules to accommodate recovery. Proper record-keeping can also help in legal proceedings or compensation.

Preventive strategies lower both the incidence and severity of workplace emergencies [37]. Risk assessments guide interventions, like engineering controls or administrative policies [38]. Rural or remote sites may rely on telemedicine to link onsite personnel with specialists [39]. Some large industries have onsite clinics to address emergencies quickly [40]. Climate change adds new challenges, such as heat stress or storm-related infrastructure damage [41]. Rising temperatures can expand the range of certain pathogens [42].

Electronic health records support continuity of care. They track exposures and help detect trends [43]. Privacy concerns remain a challenge, and workers may hesitate to share information if they fear retaliation. Balancing transparency with confidentiality is key. Researchers, clinicians, and industry leaders can develop best practices for preventing and managing emergencies [44]. International organizations publish guidelines to align safety efforts across regions [45]. Evidence-based protocols, such as advanced trauma life support (ATLS) for trauma or specialized toxic exposure procedures, reduce treatment variation [46]. However, workplaces vary widely, and clinicians must adapt to local conditions. Drills and site-specific training reveal gaps and build confidence [47].

Informal or unregulated work sectors remain difficult to oversee. Underreporting and minimal oversight lead to poor outcomes. Even regulated environments can experience “black swan” events [48]. Robust safety culture and emergency planning reduce harm. The American College of Occupational and Environmental Medicine (ACOEM) offers guidelines for acute injuries and illnesses [49]. They stress immediate hazard identification, life support, and coordination with emergency services. For trauma, controlling bleeding and stabilizing fractures is essential [50]. For chemical exposures, removing the person from the source and performing proper decontamination is key. ACOEM recommends storing antidotes and keeping poison control numbers handy [51]. Respiratory emergencies may need oxygen therapy or bronchodilators, with close monitoring for delayed edema [52].

Mental health guidelines urge early recognition of stressors, short interventions, and clear referral pathways [53]. Ergonomic guidelines address repetitive strain through hazard assessments and job modifications. Sudden back or shoulder injuries need prompt imaging and therapy [54]. ACOEM favors a team-based approach, where occupational health experts work with supervisors to limit strain [55]. Rehabilitation and return-to-work protocols must match the individual’s capacity [56]. Frequent check-ins can help catch signs of reinjury or unresolved pain. By implementing these steps, workplaces can improve safety and maintain productivity [57].

Strengthening transitions among all these areas ensures a more coherent approach to occupational emergencies. Employers, clinicians, and policymakers can collaborate to create safer environments. When accidents or exposures occur, rapid, evidence-based care preserves health and saves lives. By combining prevention with acute-response readiness, the field of occupational medicine continues to protect workers in evolving industries.

## Review

Occupational medicine addresses many acute clinical scenarios. Medical and surgical emergencies in the workplace range from minor injuries to life-threatening events. Each case usually needs fast identification of the cause and prompt supportive care. Workers may present with shock from hemorrhage, fractures from falls, respiratory failure from toxic fumes, or acute mental distress. Early intervention often leads to better outcomes. Many researchers stress a combination of strategies: engineering controls, staff education, and ready medical interventions. A single, one-size-fits-all approach cannot eliminate workplace emergencies, but combined measures can reduce the frequency and severity.

Clinicians often assess the emergency site to find immediate threats. If chemical exposure is suspected, they confirm whether inhalation or skin contact has occurred. They then arrange decontamination, supportive care, and any needed antidotes. In traumatic injuries, controlling bleeding and stabilizing fractures are the top priorities. Standardized protocols, such as triage categories, help teams deliver care quickly [58]. These protocols reduce confusion when multiple workers are hurt. Table 4 lists various workplace emergencies and first-response measures. Referring to such guidelines keeps the process orderly and can prevent secondary injuries.

**Table 4:** Selected workplace emergencies and first-response measures ABG: arterial blood gas; ARDS: acute respiratory distress syndrome

Emergency type	Immediate actions	Key considerations
Chemical exposure	Decontaminate skin, remove clothing	Use antidotes (if indicated), prevent secondary spread
Traumatic injury	Control bleeding, immobilize fracture	Rapid imaging, watch for shock
Burn (thermal/electrical/chemical)	Stop the burning process, cool or flush the area	Check for hidden tissue damage, assess the airway
Respiratory distress	Provide oxygen, remove from source	Monitor airway swelling, ABGs, potential ARDS
Mental health crisis	Remove from stressor, call for help	Assess the risk of harm to self/others, consider counseling

Employers who invest in first aid training often see better on-site outcomes. Workers trained in CPR or bleeding control can stabilize a patient before external help arrives [59]. On-site nurses or paramedics bridge the gap between the workplace and the hospital. Educational programs focus on warning signs like severe breathing trouble, uncontrolled bleeding, or altered consciousness. Swift recognition prompts immediate calls to emergency services.

Falls from height, crushing incidents, and machinery entanglements can produce complex fractures, head trauma, or organ damage [60]. Surgeons rely on imaging (X-ray, CT, MRI) to gauge the extent of injury. Some fractures call for immediate surgery, especially if there is vascular compromise. Damage control orthopedics stabilizes severe fractures in unstable patients and definitive repairs can be completed later [61].

Crush injuries may lead to compartment syndrome or rhabdomyolysis [62]. Both can cause lasting harm if not treated quickly. Fasciotomy can relieve pressure in compartment syndrome. Aggressive hydration helps prevent kidney failure in rhabdomyolysis. Delayed care increases the risk of necrosis and cardiac arrhythmias.

Burns arise from hot surfaces, chemicals, or electricity. Chemical burns typically need irrigation to remove corrosive agents. Thermal burns require cooling and an assessment of burn depth and area [63]. Electrical burns may seem minor on the surface but can injure deep tissue or the heart’s rhythm. Prompt referral to a burn unit is critical for severe cases. If hot gases are inhaled, fiberoptic bronchoscopy can check the airway [64]. Fluid management (e.g., Parkland formula) helps prevent shock.

Chemical incidents include acute inhalation of toxic gases or skin contact with strong acids or alkalis. Quick removal from the source and decontamination are vital. Specific antidotes, such as atropine for organophosphates, can be lifesaving [65]. Poison control centers guide further action. Lab tests verify heavy metals or other toxins [66].

Respiratory emergencies arise from toxic spills or preexisting lung disease. Coughing, wheezing, or chest tightness may progress to airway edema. Treatment can include bronchodilators, corticosteroids, and possibly intubation. Arterial blood gases help track oxygen and carbon dioxide [67]. Serious inhalation injuries may lead to acute respiratory distress syndrome (ARDS). Early collaboration with respiratory therapists or pulmonologists can improve outcomes.

Laboratory workers face sudden pathogen spills. Healthcare workers may experience needlestick accidents that transmit blood-borne diseases [68]. Zoonotic pathogens also threaten agricultural or veterinary staff [69]. If an outbreak occurs, contact tracing and isolation control the spread. Prompt antibiotic or antiviral treatment prevents severe complications.

Severe stress may trigger suicidal thoughts or panic. Sudden breakdowns in high-stress jobs can cause erratic behavior. Immediate counseling or psychiatric care may be required. Crisis hotlines, peer support, and early intervention reduce chronic problems [70]. Substance abuse can compound the risk of both accidents and self-harm [71].

Heat stroke occurs when core temperature exceeds 40 °C, leading to confusion and organ failure. Rapid cooling saves lives. Cold exposure causes hypothermia and frostbite, which need prompt rewarming [72]. Proper clothing, rest breaks, and hydration decrease these risks.

Confined spaces like tanks or storage rooms can have low oxygen. Entering these areas is hazardous if ventilation fails or if gas monitors are inaccurate [73]. Quick rescue attempts without the right gear often cause multiple casualties. Training and close adherence to permit rules reduce deaths.

Ergonomic problems sometimes lead to acute spinal or nerve compression. Early detection and therapy lower the chance of severe impairment [74]. Older workers or those with chronic illness may face extra risks, like sudden hypertension spikes or hypoglycemia [75]. Employers should recognize these needs and plan accordingly.

Local emergency medical services (EMS) must be aware of the facility’s layout and main hazards. Rapid transfers minimize morbidity [76]. Drills and site visits let responders practice how to stabilize patients in challenging settings. These rehearsals also highlight barriers, such as tight passages or poor water access for firefighting.

After an acute injury, rehabilitation influences long-term function. Providers often collaborate with therapists and mental health counselors. Early mobilization can shorten disability. A phased "return-to-work" helps prevent re-injury. This process must balance recovery progress with safety [77]. Table 5 highlights the core steps in a successful return-to-work pathway. Following these guidelines can reduce complications and strengthen employee resilience.

**Table 5:** Core steps in return-to-work planning

Step	Actions	Goal
Initial assessment	Evaluate physical/mental capacity	Identify limitations
Task modification	Adjust duties or schedule	Prevent overexertion/injury recurrence
Gradual reintegration	Phase tasks over days/weeks	Smooth transition to full workload
Ongoing monitoring	Track pain, fatigue, healing progress	Make timely adjustments
Final clearance	Confirm fit-for-duty status	Ensure safe and sustainable return

Prevention starts with hazard identification. Solutions can involve machine guards, ventilation, or rotating tasks to reduce strain [58]. PPE is the final safeguard. Regular audits ensure that these measures work. A strong safety culture is also critical. Workers who follow procedures and report near-misses contribute to fewer incidents [59]. Table 6 shows some common risk management steps to reduce the severity and frequency of occupational emergencies.

**Table 6:** Key risk management steps in occupational settings PPE: personal protective equipment

Step	Example	Outcome
Hazard tdentification	Evaluate tasks, tools, chemicals	Pinpoint potential dangers
Control measures	Install guards, improve ventilation	Reduce worker exposure
Training and drills	First aid, chemical spill response	Quicker, more confident emergency reactions
Monitoring and audits	Check PPE integrity, track near-misses	Identify failures and improvement areas
Leadership engagement	Allocate resources, model safe behavior	Reinforce a culture of safety

Managers set the tone by investing in equipment and enforcing standards. Technological tools (e.g., sensors and drones) can detect hazards early [60]. However, technology must be maintained and staff trained. Hands-on simulations and refresher courses ensure that procedures remain fresh in workers’ minds [61].

Occupational emergencies can affect nearby communities if chemicals escape the work site. Infectious outbreaks can spread from workplace to household [62]. Clear communication with public health authorities ensures a coordinated response if a crisis grows beyond the facility. Global operations must adapt to varying safety regulations, supply chains, and cultural norms [64]. Unified company policies still rely on the same fundamental steps of hazard control and rapid response.

Thorough reviews of emergencies highlight technical failures, human errors, and leadership gaps [65]. A blame-free environment encourages honesty and drives improvement. Sharing findings with industry partners helps others avoid similar incidents. Keeping strong safety measures in place also cuts costs over time, since preventing accidents is cheaper than handling their aftermath [66].

Occupational health teams-physicians, nurses, industrial hygienists-bridge clinical care and workplace operations. They track patterns, update protocols, and advocate for worker well-being [67]. Psychosocial aspects also matter. High-strain settings raise the risk of errors or near-misses [68]. Future research may refine best practices and explore emerging technology, but strong fundamentals remain vital: training, routine maintenance, and fast clinical response [69].

Environmental concerns, such as oil spills or waste disposal, carry secondary exposure risks for workers [70]. Safety engineers, industrial hygienists, and health experts must cooperate to protect both employees and the environment. Conflicts can arise over resource allocation, but all must agree on safeguarding human health. Modern companies may hire specialized roles (e.g., ergonomists or occupational psychologists) to address specific hazards [71]. Communication systems must also include backups for power outages or network failures [72].

Economic downturns can push employers to cut safety budgets, raising accident risk. Global crises may disrupt supply chains, limiting access to PPE [73]. Flexible, well-planned occupational health policies can handle these pressures. Community partnerships and active participation from leadership keep the workplace resilient.

Occupational emergencies span trauma, burns, chemical exposures, respiratory distress, infectious hazards, psychological crises, and more. Rapid identification, immediate care, and coordinated efforts with EMS are critical. Return-to-work programs, targeted training, and a culture of safety can significantly support recovery and reduce repeat incidents. Ongoing research, new technology, and collaboration among stakeholders will continue to shape best practices. Yet the core principles remain consistent: prevention, early detection, and prompt medical intervention.

## Conclusions

Many occupational emergencies can be prevented with systematic hazard assessments, targeted training, and consistent use of protective equipment. Rapid recognition of urgent symptoms, along with standardized triage protocols, helps clinicians stabilize injuries and reduce complications. Quick evacuation in cases of chemical leaks or fires also lowers the risk of secondary harm. Evidence shows that supportive workplace cultures encourage near-miss reporting, which reveals unseen threats and promotes proactive interventions. Early involvement of mental health services protects workers from lasting stress-related disorders. Rehabilitation programs and phased return-to-work plans reduce disability and support long-term recovery. Progress also relies on updated regulations and technology to detect and manage new hazards as industries evolve. Coordination among clinicians, employers, safety experts, and policymakers remains key to ensuring safer work environments.
